# Fractal properties and morphological investigation of Nano hydrochlorothiazide is used to treat hypertension

**DOI:** 10.1186/s40360-018-0259-5

**Published:** 2018-11-09

**Authors:** Amir Kazemi Korayem, Shahriar Ghamami, Zahra Bahrami

**Affiliations:** Department of Chemistry, Faculty of Science, Imam Khomeini International University, Qazvin, 34148-96818 Iran

**Keywords:** Fractal, Cardiac, Hypertension, Hydrochlorothiazide, MATLAB, Drug delivery

## Abstract

**Purpose:**

High blood pressure (hypertension) is a relatively common condition that increases blood pressure in the arteries abnormally, causing problems such as heart disease and stroke. Blood pressure is a force that is felt through the walls of the blood vessels and has a direct relationship to the power of pumping the heart and vascular resistance to the blood flow. One of the lowing of blood pressure medications is hydrochlorothiazide (Hctz) which is used to treat high blood pressure and swelling from heart failure, liver damage, and other minor actions.

**Procedures:**

This study explains the effectiveness of Hctz with the help of “fractal dimension”. To perfect investigate the fractal dimension, we used the Hctz drug nanoparticle form then using MATLAB software, homogeneity levels or heterogeneity of Nano Hctz using SEM images were computed.

**Results:**

We calculated histogram plot with SEM image by MATLAB software which that its standard deviation was eclose to zero and it can explain that the scattering of data is low and their amount is in one suffering. Fractal dimensions obtained from Matlab and SPSS software for normal distribution, correlation, standard deviation, mean, cumulative frequency and variance analysis were analyzed.

**Conclusions:**

In this research, the association between Hctz treatment effects with the point of view of the fractal dimension of the drug was demonstrated to prove the properties of the drug in the body. in the near future, drug fractal studies can improve the development of new drugs and treatments with minimal cost than clinical approaches by linking chemistry, mathematical sciences and pharmaceutical sciences.

## Introduction

**Fig. 1:**
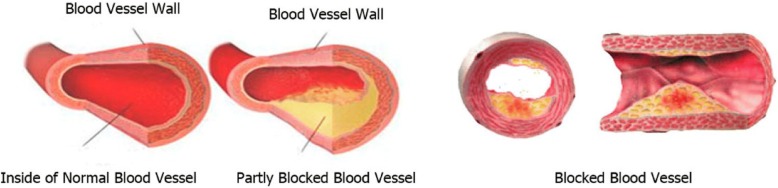
Hypertension happens when the force on the walls of blood vessels from the blood within them is more than normal

High blood pressure or blood pressure is one of the most important causes of heart disease and stroke worldwide [1–3]. According to available statistics, about 20% of the adult suffer from high blood pressure [4]. Blood pressure in men is twice that of women [5, 6]. The number of people with high blood pressure is expected to rise to 1.56 billion people worldwide by 2025 [6, 7]. Among the illnesses, blood pressure is known as a silent killer because it has no warning signs, and many people are not aware of it [8–10]. The increasing prevalence of the disease is attributable to lifestyle and dietary factors such as physical inactivity, acute stress, intensive exercise, alcohol and smoking, and a high sodium diet (usually from Processed and fatty foods) is shown in Fig. 1 [11–16]. Blood pressure increase in the morning. When blood pressure is measured in the morning, high blood pressure is associated with an increased risk of stroke and heart disease [17]. Drugs are generally started as monotherapy (just one drug) and at a low-dose [18]. Side-effects associated with antihypertensive drugs are usually minor [19–21]. Hctz is one of the drugs used to treat high blood pressure [4, 22].

Geometric shapes (circle, triangle, square, straight line, polygon, etc.) are quite familiar with Euclidean geometry, which actually help us to better classify our surroundings. However, the truth is that the visible nature does not have an Euclidean geometry, in other words, the real world shapes are not Euclidean! In fact, the geometry that can describe our world is called fractal geometry [23]. In fact, fractals have three characteristics from a geometric point of view:

1. Self-similar.
2. On a small scale, they are complex.
3. Dimension is not an integer (for example, 1.51).

But fractal calculations:

Contrarily, fractals do not have correct dimensions. When the dimension of a fractal it is said to be 1.2, it means that it is more complex than a line, and simpler than a page. Calculation of this dimension is obtained after a series of logarithmic formulas [24]. If the dimension along a line is equal to one, then the dimension of a page is equal to two, and the space dimension is three. An example of the fractal is presented herein using math intuition.

The Koch Snowflake!

Consider the equilateral triangle. Step by step:

- Draw an equilateral triangle.
- Divide each side into three equal parts.
- Draw an equilateral triangle on each middle part.
- Divide each outer side into thirds.
- Draw an equilateral triangle on each middle part.
- Repeat until you’re satisfied with the number of iterations.

**Fig. 2:**
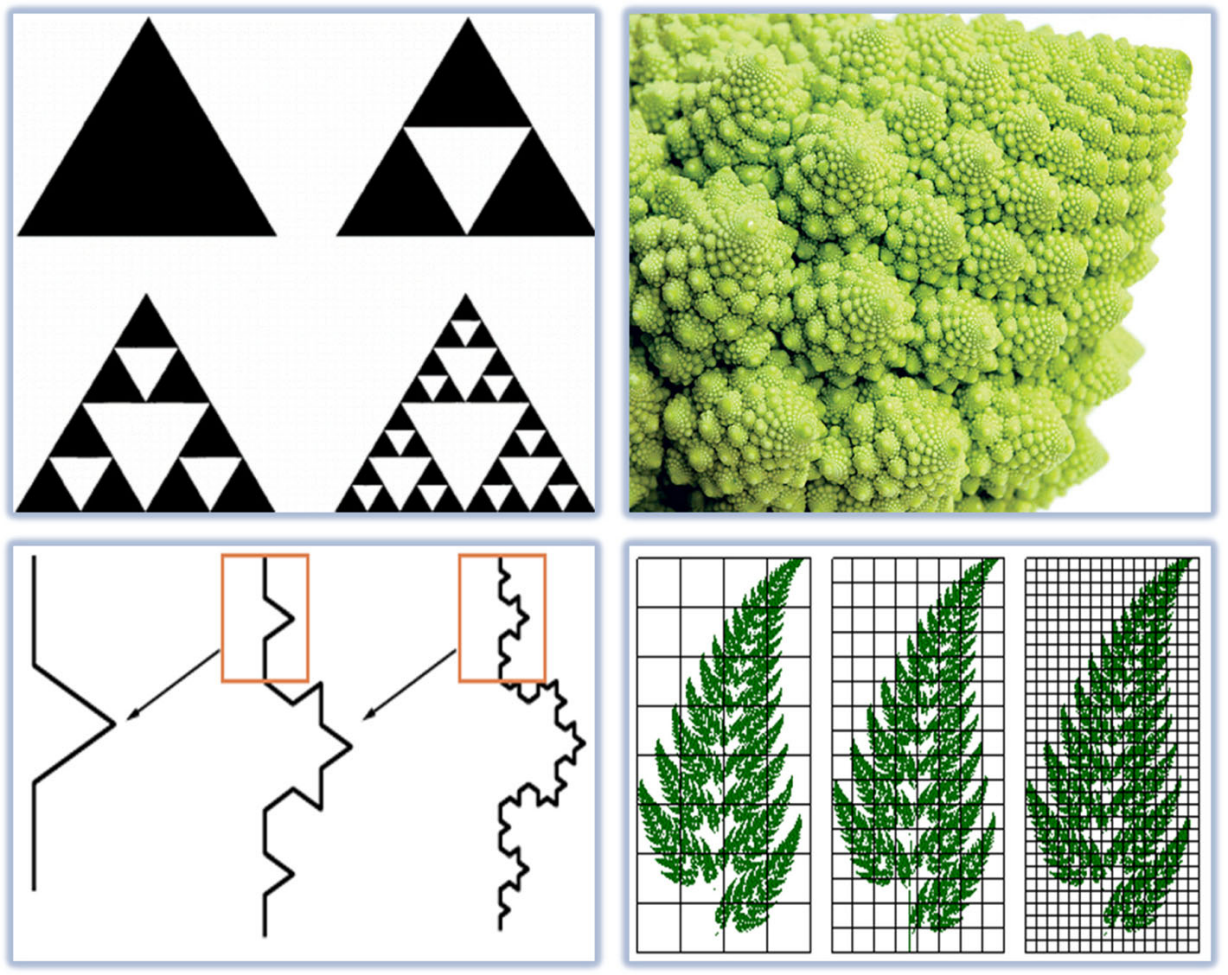
Example of branching fractals, Selfsame Fractal Zoom-Zones, showing the structural elements, or elementary geometrical units

Decorate your snowflake how you like it. Regularly, it is performed repeatedly to have fractal shapes (Fig. 2).

In nanoscale, many self-assembly processes create fractal patterns. Fractals are shapes that are repeated in many magnifications and are common throughout nature and appear in the natural environment, biological systems, and human physiology. As a biological example, the dendritic structure of the human neuron is a nanoscale nerve structure and uses this Fractal geometry connectivity to generate advanced information processing [25, 26]. The same connection can also be used with the use of artificial fractal electrical circuits for future commercial computers. By combining nano and fractal, many of the surrounding events can be justified. Regarding nanotechnology, drugs can with improved physical and chemical properties, such as high reactivity and the ability to select a cell membrane from pathogenic bacteria, can be produced [27]. Many fields are exciting at the start of this journey to improve the basic quality of human life. Fractal Property is a physical characteristic that is expressed at the supermolecular level, on a microscopic scale and on a macroscopic scale. To calculate fractal dimensions, image processing is performed in divided windows using MATLAB. This software can display a binary image with the characteristics of fractal nanoparticles. Fractal Geometry has recently been recognized as an analytical tool suitable for describing nanostructures. Also, fractals are infinitely complex models that are self-similar across different scales [28, 29]. The fractal dimension parameter is determined by calculating the predicted perimeter length by taking steps of a known length along the border and including the number of scales [30, 31].

One of the good applications of the fractal can be the development of anticancer drugs and this drug discovery. Cancer tumor cells exhibit irregular, heterogeneous, and very distinct structures, and therefore we have difficulty in making effective anticancer drugs [32, 33]. Fractal models can be used in nonhomogeneous systems and non-Fickian processes in pharmacokinetic models, such as cancer drugs [34].

## Experimental

### Materials and methods

Starting materials were obtained from Merck (Berlin, Germany) and used without further purification. The surface morphology of the product was characterized by a field emission scanning electron microscope (FESEM) (Hitachi S 4160, Japan) with an accelerating voltage of 20 kV. The size of selected samples was estimated using the Scherer method. For identification, FESEM samples were coated with gold. This is essentially a function in MATLAB (MathWorks, Natick, Massachusetts, USA) as a powerful software application widely used for data design and analysis e, programming and execution of engineering and research calculations. Thus, MATLAB data were analyzed with statistical packages to discuss statistical aspects and provide comparable results from this study (SPSS, IBM, Armonk, NewYork, USA). To obtain quantitative and qualitative information related to each image in addition to RGB, Histogram MATLAB scales were determined for the images, which shows the symmetry and/or asymmetry as well as the uniformity/non-uniformity between points on the image. The graphs determine differences between different parts of an image.

### *Synthesis of Nano Hctz (*C_7_H_8_ClN_3_O_4_S_2_*)* is used to treat hypertension

**Fig. 3:**
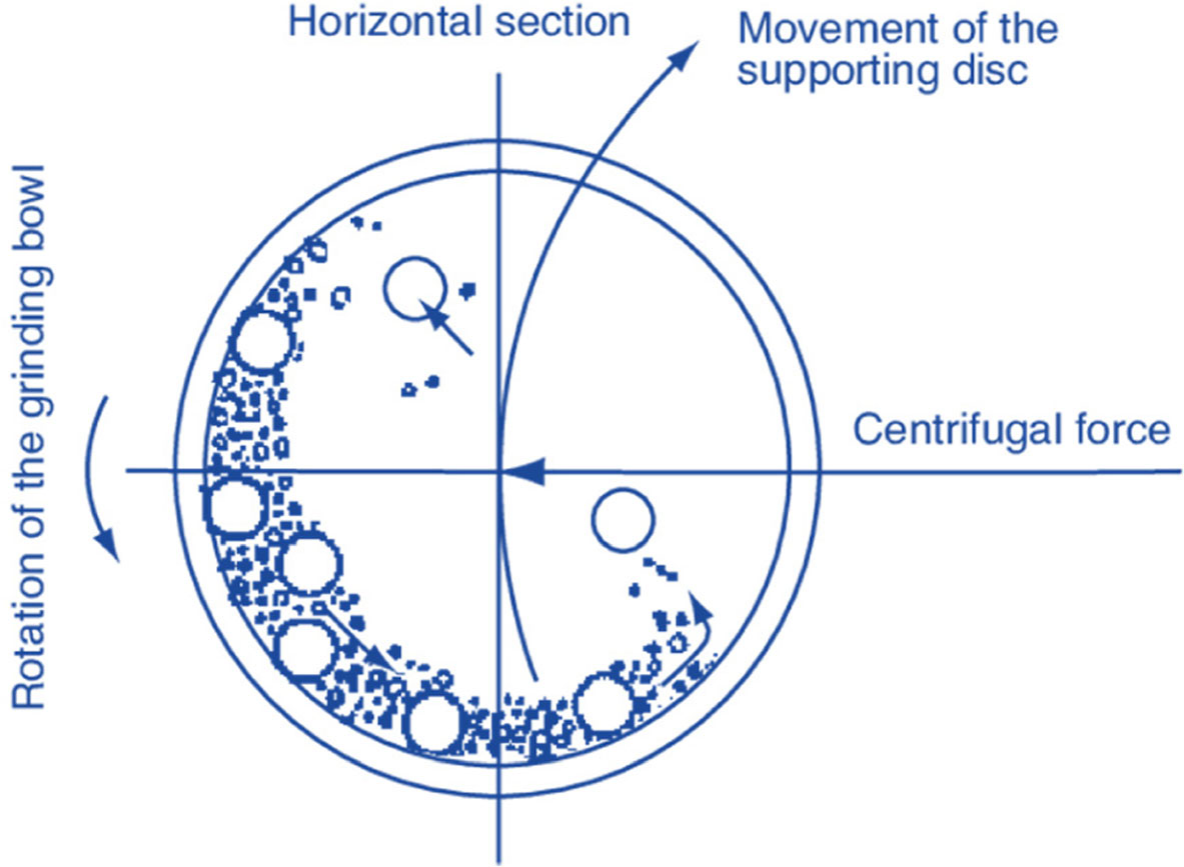
Planetary ball mill process

Nanoscale particles were produced using the “top-down” method in this study. In this method, the particles are miniaturized by grinding to a nanometer size. The appropriate tool for this method is a ball mill process (Fig. 3). In order to make a nanoparticle in a ball mill, the grinding jar and balls should be made of an abrasion-resistant material such as ZrO_2_ to minimize contamination of the sample material by abrasion. In this device, the rotation of the chamber and a plate around a single axis creates a force from the center that can reach 20 times the acceleration of gravity [35]. The drug was first placed in an oven at 70 °C for 1 h. Then, the dry drug was poured into a mill cup containing 15 different-sized metal-polymeric balls. It took around 5 hrs to become a nanoscale [36, 37].

### Computational method

Twenty-five sections of SEM images were selected and their fractal dimensions calculated using MATLAB software. Then, fractal computations were transferred to SPSS software to analyze normal distribution, correlation, standard deviation, mean, cumulative frequency and variance [38]. Given the resulting numbers, we drew a logarithmic diagram in which the vertical and horizontal axes were log (1/*r*) and log *Nr*, respectively, and Coefficient of the size as the box was 1.3. We used a program written in MATLAB to calculate the fractal dimension [39]. There are several methods for calculating the fractal definition, including (1) the self similarly definition [40], (2) the mass fractal dimension, (3) the spare-sphere fractal dimension, (4) the Euclid dimension, (5) the grid style, (6) an analytical approach for fractal diminishing, and (7) the box-counting fractal dimension. In this study, the self-similar method was used to determine the fractal dimension of FESEM images, which was analyzed using the box-counting method [41–43]. A variety of methods are used to estimate fractal dimensions. Due to the ease of mathematical calculation and empirical estimation, boxthe -counting dimension is one of the most widely used dimension assessment to determine the fractal dimension of different phenomena. The characteristics of this method include ample algorithm, a high precision with a relatively smaller grid selection, and usability in higher pixels. The fractal dimension was calculated theoretically by applying the following formula:

1 $$ \mathrm{D}=\underset{\mathrm{r}\to 0}{\lim}\frac{\log Nr}{\log \left(1/r\right)} $$

Where *D* is the fractal dimension and *N* *r* is the number of squares including a part of the considered fractal dimensions [44]. For the box-counting fractal dimension, we assumed that *F* is a nonempty and bounded subset of *Rn* and we supposed that (*F*) is the fewest number of collections witha maximum dimension of (*r*) that can cover (*F*). The box-counting dimensions under and above (*F*): if they are equal we say they are equal to the box-counting dimension or the *F* box dimension; then we define:

2 $$ \underset{\mathrm{B}}{\mathrm{dimF}}=\underset{\mathrm{r}\to 0}{\lim}\frac{\log Nr(F)}{-\log (r)} $$

The circle of cubes in r coordinates from Rn implies cubes in the following form:

If [m1r,(m1 + 1)r] × ··· × [mnr,(mn+ 1)r], then mn,...,m1 are integers.

Finally, Nr√(F) ≤ Nr‘(F).

If r√n < 1,

Then

3 $$ \frac{\mathrm{logNr}\surd \left(\mathrm{F}\right)}{\log\ \left(\mathrm{r}\surd \mathrm{n}\right)}\le \frac{{\mathrm{logNr}}^{\hbox{'}}\left(\mathrm{F}\right)}{\left(-\log \surd \mathrm{n}-\log\ \mathrm{r}\right)} $$

If we limit ${\dim}_BF=\frac{\lim }{\mathrm{r}\to 0}\frac{{\mathrm{logNr}}^{\hbox{'}}\left(\mathrm{F}\right)}{-\log\ \mathrm{r}.}$

Then

4 $$ {\dim}_BF\underset{\kern1.25em \mathrm{r}\to 0}{\le \lim}\frac{{\mathrm{logNr}}^{\hbox{'}}\left(\mathrm{F}\right)}{-\log\ \mathrm{r}} $$

Finally: *N*_*r*_^'^(*F*) ≤ 3^*n*^*N*_*r*_(*F*).

## Results

### Analysis of the effective factors for creating the Nanodrug

**Fig. 4:**
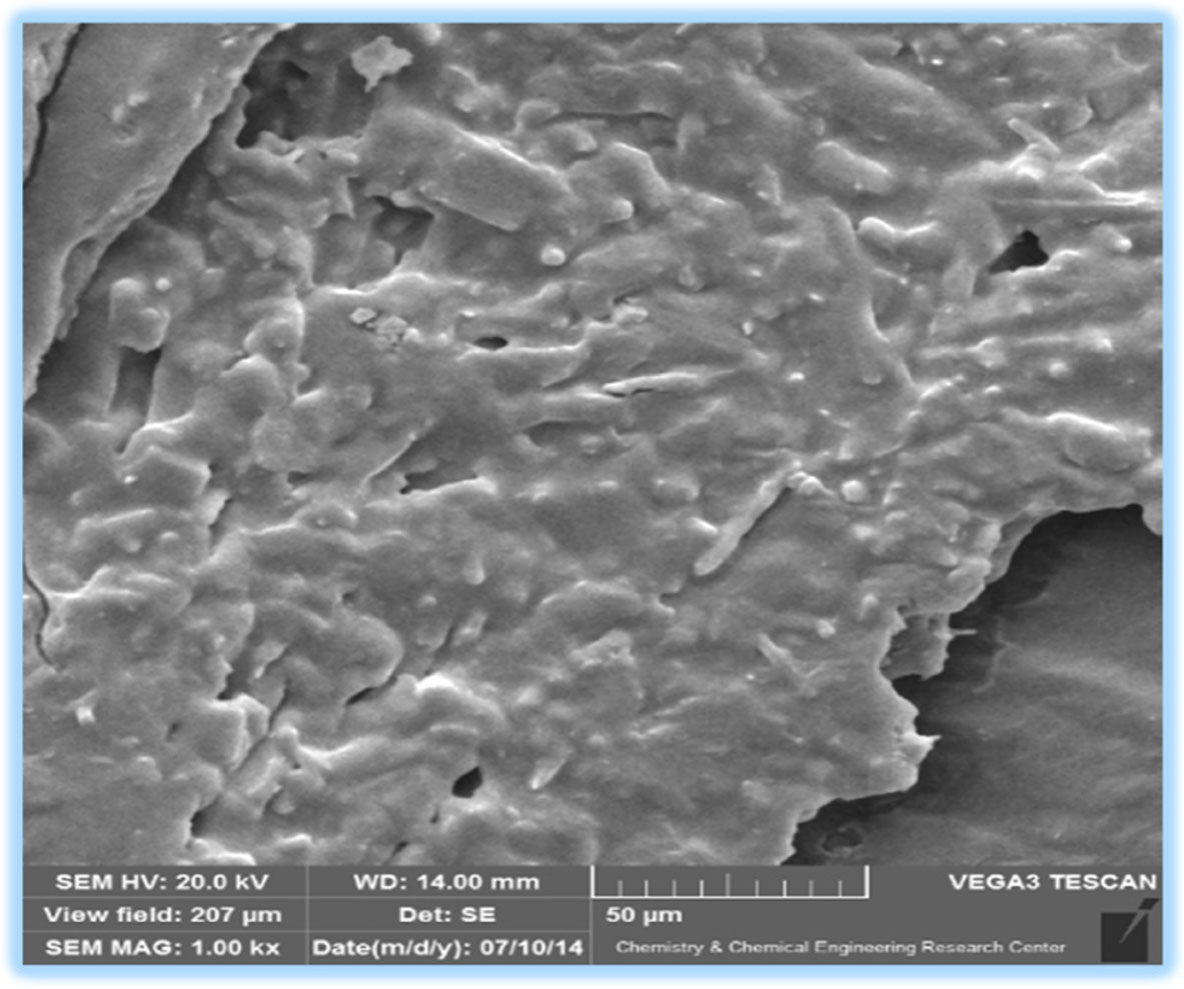
SEM images of hydrochlorothiazide

It should be noted that the ball milling technique (as one of the high-to-low methods) was used to make nano Hctz [45]. which was confirmed through the analysis of SEM images taken from the sample. The SEM image (Fig. 4) shows that the particles are less than 100 nm confirming the formation of nano Hctz. Then, fractal dimensions were calculated 20 randomly selected SEM images using MATLAB software. For this fractal dimension, the standard image, average, maximum and minimum range histogram, asymmetry and also harmonics were obtained using SPSS software [46, 47].

### Morphological properties

The images are taken from the outer structure and the morphological structure of the sample show that the nanopowder is made of very dense, porous and cavity holes, and its spherical particles are below 100 nm; The larger particles observed in the images, in fact, are the accumulation of smaller particles that appear in an irregular spherical shape [48]. The SEM images of the Nano Hctz are shown in Fig. 4. cumulative percentages are a simple way to compare different sets of data.

### Analysis using MATLAB and SPSS data

**Fig. 5:**
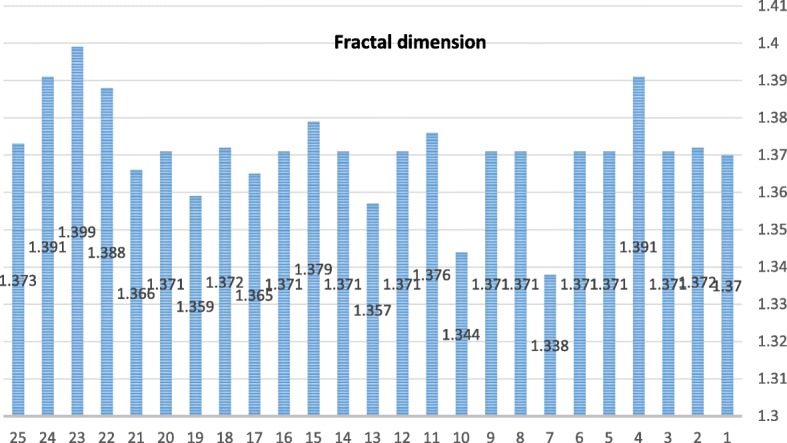
Statistical values of the selected Hydrochlorothiazide nanoparticles

The statistical and numerical interpretation of fractal dimensions is given in Table 1. According to FESEM images, fractal dimensions separated from the image are calculated. With the point of view, sometimes when the same value appears more than once, it is repeated as the frequency of a known number. The cumulative frequency was used to determine the number of explanations below or above a specific value. The total frequency of all modules calculated below the upper-class class is the cumulative frequency of that class, which adds a class interval to the frequency and frequencies of the first distances to the distance of that class. This is a different method for expressing frequency distribution. Figure 5 shows mean (1.919), median, maximum (1.399), minimum (1.338), range (0.94), variance (0.000174), standard deviation (0.013209), and skewness obtained for fractal dimension data using SPSS software.

**Table 1:** Chart analysis of fractal dimension boxes Hydrochlorothiazide

Log(res)	Log (boxnum)
0	9.93
0.6931	8.898
1.386	8.021
2.079	6.978
2.773	6.112
3.466	5.157
5.545	2.293
6.238	1.339

## Discussion

**Fig. 6:**
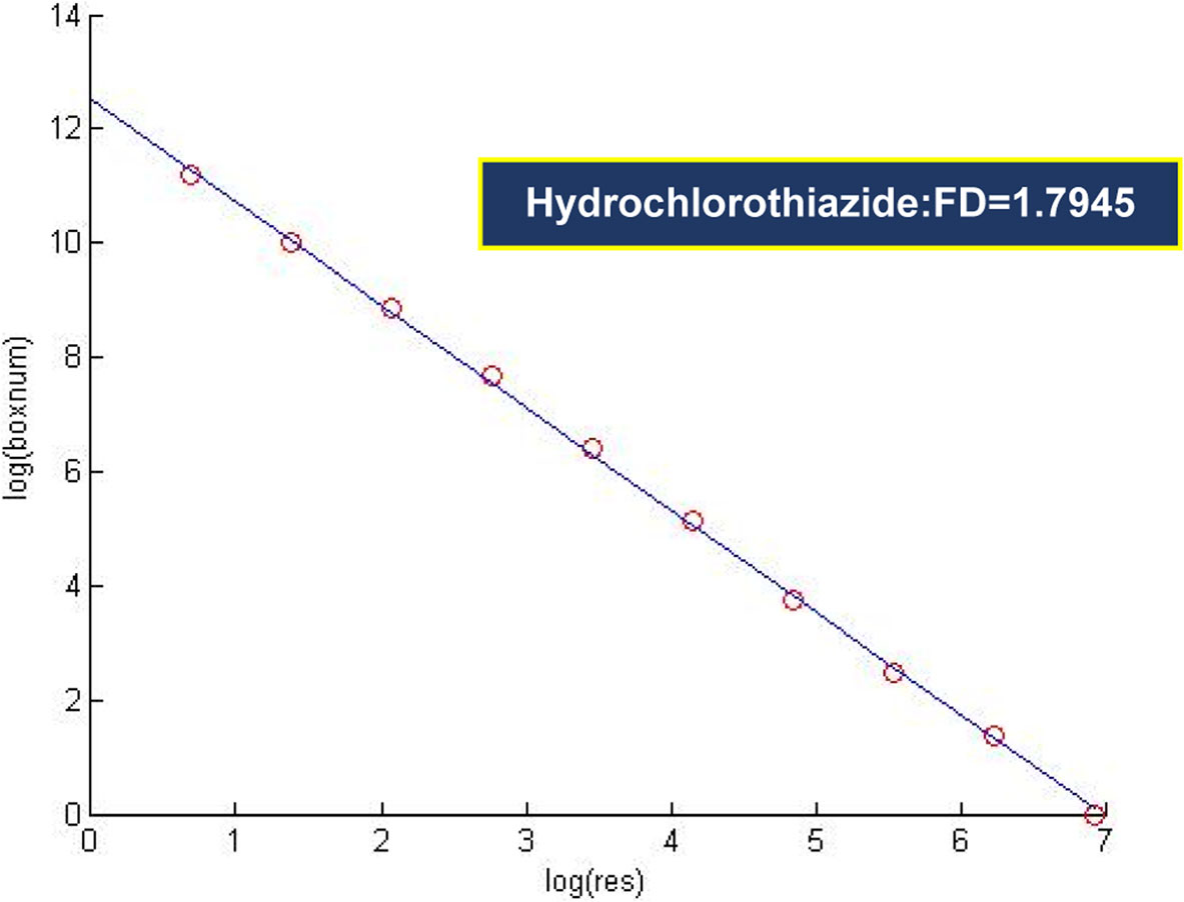
Chart analysis of fractal dimension boxes hydrochlorothiazide

There is a large deviation from the normal distribution in the assumptions of normal errors, all of which are shown by statistical calculations and calculated intervals for regression, and its value can have understandable effects on the accuracy of the results. A simple method for controlling error assumptions will normally be distributed if the likelihood of a probability fraction (PP) is correctly recognized by the software, also if it is accepted that the line is based on a normal assumption. The numerical value of RGB is calculated in the same amount of porous holes in dark and bright images. In the images of all images, dark areas with numbers less than 0.5 and 0.5 points are clearly displayed with more. Figure 6 shows the calculated dimension of nano box Hctz.

The distribution of pp. was obtained using a statistical statistic of the data. According to the results, the data on a line indicates that the population is normal. Nano-fractal dimensions of the drug were calculated as 1.371. The location was found anywhere in the chart using the MATLAB software (Table 1). Input (res) or X is an independent variable in this graph. This factor represents 1 / r, the size of the box image. Login (box num) is an associated variable or Y, which indicates the number of boxes (box-counting method) to obtain the position of these points. Obviously, the difference for each image in the log (box num) will produce a fractal pattern in each selected image. Regardless of the unrestrained and orderly images, a special order or pattern is carefully obtained in the figures.

The correlation coefficient between the points’ data (Table 1) in our software SPSS was obtained using Pearson’s correlation coefficient.

After obtaining the correlation coefficient, data were used for linear regression analysis of the charges (Table 2). The line slope was calculated narrowly as 1.371 with MATLAB and then computed by the software. Charts intercept was 9.892. Table 3 represents the obtained maximum and minimum values as well as uncertainty data.

**Table 2:** Correlation coefficient calculated between the points on the graph nano hydrochlorothiazide

Correlations*	LOGres	LOGboxnum
LOGres		
Pearson Correlation	1	−1.000
Sig. (2-tailed)		.000
N	8	8
LOGboxnum		
Pearson Correlation	−1.000	1
Sig. (2-tailed)	.000	
N	8	8

*Correlation is significant at the 0.01 level (2-tailed)

**Table 3:** Regression coefficient nano hydrochlorothiazide

Coefficients ^a^ Model		Unstandardized Coefficients		Standardized Coefficients	t	Sig.
		B	Std. Error	Beta		
1	(Constant)	9.892	.023		432.537	.000
	LOGres	−1.371	.007	−1.000	−207.737	.000

^a^ Dependent Variable: LOGboxnum

The linear equation was obtained for the nano-medicine using Table 4.

$$ \mathrm{Log}\ \left(\mathrm{box}\ \mathrm{num}\right)=-1.371\ \mathrm{Log}\left(\mathrm{res}\right)+9.892 $$

**Table 4:** Calculate the number of maximum, minimum, average and uncertainty of data related to nano hydrochlorothiazide

Residuals Statistics ^a^	Minimum	Maximum	Mean	Std. Deviation	N
Predicted Value	1.34056	9.89152	6.09100	3.047221	8
Residual	−.063656	.038483	.000000	.035931	8
Std. Predicted Value	−1.559	1.247	.000	1.000	8
Std. Residual	−1.640	.992	.000	.926	8

^a^ Dependent Variable: LOGboxnum

As you can see, placing data on a straight line indicates that this is a normal population.

**Fig. 7:**
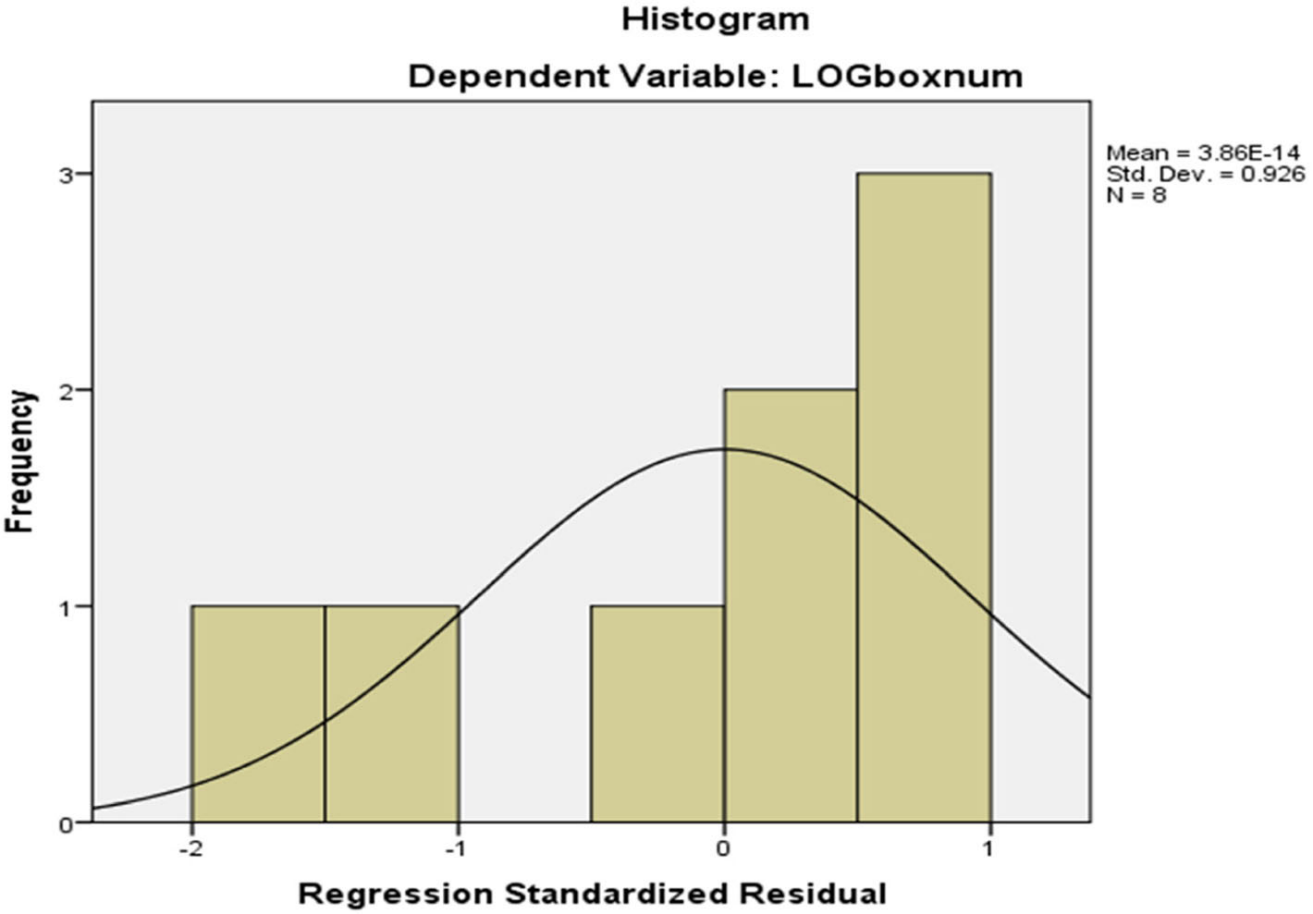
Histograms of the nano hydrochlorothiazide

In RGB images, the brightness of each part of the image was processed and matched with MATLAB software. According to RG tables and images, none of them larger than half are not characteristic of the bright points of the image, while none of the half RG of the image is displayed in the image. RG Distribution of different points of the image, shown in Fig. 7.

## Conclusion

The primary purpose of this study is to prove the fractal behavior of Nano drugs Pharmacy and Medicine Knowledge. In this research, after nano-making of a Hctz drug, the fractal dimensions of the nanoparticle were calculated and the results were entered into the SPSS software for statistical analysis. Subsequently, using MATLAB software, homogeneity levels or heterogeneity of Nano Hctz using SEM images were investigated. After calculation, it was found that the produced particles are not homogeneous and their size and morphology are different. The results show that the differences in magnification and reduction of SEM images cause distinctions in fractal dimensions. The nano Htze fractal indicates that its standard deviation is close to zero, and if the standard deviation of the data is close to zero or exactly zero, then it can be argued that the scattering of data is low and their amount is in one suffering. In scientific studies, data with a difference of more than two standard deviations from the mean value are usually considered as offset data and removed from the analysis. Some of the properties of Hctz, such as the beneficial effects of therapy, drug delivery, and medication degrees in the body, can be proven by studying and calculating fractal behavior, A research from this perspective, which was done for the first time, can be extensible for other drugs in medicine. We believe that in the not too distant future, fractal studies will be an effective tool for classifying drugs, diseases and tumors and will create a new horizon for disease prevention and treatment. Obviously, this type of study is much Cheaper and less risky than clinical studies.

## Abbreviations

Hctz: Hydrochlorothiazide
SEM: Scanning electron microscope
